# Was It Wise for the American Medical Association to Change Its Code of Ethics?

**Published:** 1903-12

**Authors:** D. W. Cathell

**Affiliations:** Baltimore, Md.


### SELECTION.

### Was it Wise for the American Medical Association to Change its Code of Ethics?

By D. W. CATHELL, M. D., of Baltimore, Md.

AT THE last annual meeting of the American Medical Association, held at New Orleans, May, 1903, its old code of ethics was changed by the following action: The Special Committee on "Revision of the Code of Medical Ethics," composed of Dr. E. Eliot Harris, Dr. William H. Welch, Dr. J. D. Bryant, Dr. Nicholas Senn, and Dr. T. J. Happel, after agreement with a larger committee, composed of one member of the House of Delegates from each state, submitted a preamble that provided that whatever might be the form of the document adopted to replace the old code, its sphere should be suggestive and advisory only, and not compulsory. This preamble was unanimously adopted.

The same committee then submitted a body of medico-moral precepts entitled, "Principles of Medical Ethics." The Association, through its house of delegates, also promptly adopted these principles by a unanimous vote, which was followed by great and prolonged applause, showing complete unanimity on the subject, and today one of the questions before all thoughtful medical men is: Was it wise for the American Medical Association to change its code of ethics?

That this change inaugurates a better and wiser policy for the profession of our country, I am quite sure, for the following reasons: although the moral sentiments of the old code had done good in the early years of its existence, by binding our profession together and drawing a line that excluded many unworthy persons from recognition, yet the conditions that then existed have gradually changed, until now our only opponents of any number are the homeopaths, a body of educated men, physicians under the laws of the various states, men of good professional character, correct morals, and much esteemed by those who know them best. In dealing with such men the old method of 1847 was no longer the wisest plan and chiefly on this account from these principles, the nonconsultation clause was omitted, thus relagating to each and every physician the right to follow the guiding impulses of his own judgment whenever the important subject of consultation arises.

The reason why we could not previously consult with outsiders was that since 1847, consultation with them was specifically forbidden by the code of ethics of the American Medical Association, and every member waived his natural right to do so when he joined that body or any subordinate society. This restriction being now removed, every individual's right to consult with whom he pleases is again restored, and our new principles encourage the exercise of this right in the following words: "The broadest dictates of humanity should be obeyed by physicians whenever and wherever their services are needed to meet the emergencies of disease or accident."

Among the chief reasons for this change from "code" to "principles," was that the old code's authority and its usefulness had decreased year after year until many of the best of our number had become either lukewarm or hostile toward it. Many good men have never even read it, and everybody knew that it no longer stood as an honored and efficient guide Besides, the chief evils it was intended to correct had either disappeared or got beyond its reach. Indeed, had its boycotting features been set aside forty years sooner, it would have saved our profession many dilemmas and many mortifications, for medical boycotting is a bad thing when it does not work, and the events of these forty years have proved that it was an unwise method in dealing with the homeopathic portion of our foes, and one that had a directly opposite effect from that which was intended—namely, to exclude all who held to Hahnemann's or anybody else's dogmas from membership in our societies, and thereby prevent dogmatists from using them to gain a foothold with the public. This all our enemies forthwith stigmatised as "persecution," and we were at once everywhere denounced as "allopathic persecutors."

I shall surprise you by the following statistics of what their battle against this so-called "persecution," has done for our homeopathic brethren in the United States. The medical profession of all other nations protested against or laughed at it, while we were the only country in the world which built a "Chinese wall" against them.

The *North American Journal of Homeopathy,* for September, 1903, tells us that in the year, 1900, there were in the world 10,635 homeopathic physicians distributed as follows: In Great Britain and Ireland 201, or one in 203,000; in France 211, or one in 192,-000; in British America 87, or one in 64,000; in Uruguay 7, or one in 133,000; in Switzerland 22, or one in 151,000; in Australia 29, or one in 155,000; in Spain 118, or one in 163,000; in Belgium 41, or one in 164,000; in Holland 17, or one in 300,000; in Denmark 8, or one in 306,000; in Mexico 32, or one in 423,000; in Brazil 33, or one in 428,000; in Barbadoes 5, or one in 38,000; in Italy 42, or one in 772,000; in Argentina 6, or one in 809,000; in Russia 66, or one in 1,608,000; in Portugal 3, or one in 1,674,000. There were also 14 in all India, 2 in all China, 1 each in all Cape Colony, in all Sweden, and in all Venezuela, as well as 3 in the Hawaiian Island and 1 in Alaska. Also, in Germany, where both Hahnemann and homeopathy were born, he in 1755, and it about 1790, there were in the year 1900, in all Germany, only the ridiculous number of 290, or one to every 194,000 inhabitants, while in our United States of America, where our profession adopted a nonintercourse policy almost as soon as homeopathy had reached our shores, we had in 1900, no fewer than 9,369 persons who called themselves homeopaths, or 1 to every 8,000 inhabitants—88 per cent. of all in the world; all fat, all thriving, and all fervently hoping that our good old prohibitory clause would long continue to assist; but, alas! for their hopes, this was the very knot on which our Alexanders used their sharpest swords in May last at New Orleans.

We, of today, can easily see that if in the beginning, instead of making this question a cause for rejection or expulsion, our predecessors had welcomed into fellowship such of these men as were educated and lawfully qualified, homeopathy would never have been raised into the dignified position of a rival school, and we would never have had to ask the public to choose between them and us; because they could not have raised the cry of "persecution," and without this there would be today no existing homeopathic medical societies, no homeopathic medical colleges, no homeopathic medical journals, no homeopathic hospitals, and the puzzling question how to deal with them would not be now confronting us. Even had we done this 30 years ago, neither the homeopaths, nor the eclectics, nor any other important rival would now exist, except in the forgotten pages of history.

This change of base is the most momentous step the American Medical Association has ever taken, for it removes all the restriction that separates the ins from the outs in consultation, and gives back to our specialists, our surgeons, our professors, and all other medical men, the right to consult, or not consult, with Dr. Heterodox, Dr. Homeopathy, Dr. Eclectic, or Dr. Anybodyelse, whenever any sufficient motive prompts. In such consultations we nonsectarians can hereafter meet our rivals face to face everywhere, whether in city, town, village, or rural district, and incidentally show our superiority where we are superior, fearlessly depending for the final solution of the great question of KIND, on the irrefragable law of the survival of the fittest.

But, Dr. Doubter or Dr. Oldcode may ask: If the Principles of Medical Ethics have no binding force, what is their use? My reply would be: These principles are intended to give correct and uniform suggestions and advice to the host of new members who are constantly entering the profession, and to all others who may feel the need of their admonishing precepts.

This being their only function, in my opinion, a better title and one that would plainly show to the whole world their source of origin and their nonproscriptive character would be: *"Ethical Principles that should Govern the Medical Man."* FORMULATED BY THE AMERICAN MEDICAL ASSOCIATION. This question I respectfully submit.

I also believe their features should be improved and made more modern by expurgating the paragraphs on Visits to the Sick, Respect to Seniors, Tenderness to Patients, Arbitration in Consultation, Attending One's Own Family, and the like, and adding tenets more abreast of twentieth century conditions. The document should also be simplified by abolishing the ancient and useless chapter arrangement, and the subtitles, and by rearranging and numbering its various paragraphs.

Now, barring the consultation question, what did our governing code do for us during its last 25 years that the unwritten law that governs gentlemen could not have done? Or I will put the thought in a different way: Can anyone show me a medical man who, previous to the seventh day of last May, could be depended on to do the right thing with a code hanging over his head, who cannot now be depended on to do it without one? No, because no code can specify unfailing rules for being a gentleman or put correct principles in anyone's heart if the instinct is lacking. On the contrary, so far as my observations extend, ungentlemanly acts are far less frequent in all circles in which a sense of personal honor is the incentive; and if this be so, individual responsibilities for personal deportment will henceforth be felt among medical men more strongly than ever, and a more refined and gentlemanly intercourse will surely follow.

The paragraph of the code interdicting consultation with outsiders was always contrary to the judgment of the press, the bar, the pulpit, and the people, and all our arguments were never able to efface the general belief that we were a narrow-minded and illiberal sect who held *ethics* above humanity, and no one ever assigned "*the code*" as a reason for refusal to consult, that convinced a suffering man or anybody else that this restriction was not brutal and unfeeling, and it was not our code's fault that a larger portion of the public did not angrily desert from us to "the persecuted."

Above all else, this change of base frees us from the old charge of bigotry, and brings up new and nobler issues, for this was the vulnerable spot in our armor at which our enemies of every kind directed their partisan attacks. Indeed, nowhere in the annals of human invective is recorded a more vehement, or a more continuous, or a more successful attack than the combined armies of all these "persecuted martyrs" made on this paragraph year after year, with the public as judges, with "allopathy," "old school," "crucifixion," "persecution," "jalap," "calomel," "salivation," "bleeding," "manslaughter," and a whole hatful of other fulminating nouns and adjectives added to make it expressive. But all their literature was as nothing compared to the thousands of unfair and partisan tales told at the bedside, in the parlor, and on the curbstone to every man, matron and maiden who would listen to their harangues.

They even carried their attacks into all public questions and contested for army appointments, naval appointments, and political appointments; everywhere crying "persecution;" and so deeply did their missiles penetrate the public heart that some of those who were lured away from us 40 years ago are today a hundred times more bitter toward "allopathy" and the "allopaths" than their medical attendants are, and would trust their lives to an empty-headed outsider a thousand times sooner than to the best "allopath" in the world. And there are at this moment people in every large community who would almost rather die under the care of an irregular than to get well under the hands of a regular. Why? Chiefly, because that paragraph shut out the eclectics, homeopaths, thompsonians, and others, and kept them incessantly appealing to public prejudices, and in this way the code acted constantly and powerfully in favor of the very ones it was intended to destroy. These terrific attacks on us caused hundreds of thousands of valuable lives to be entrusted to irregular care, and millions of dollars to go into their pockets, while our clansmen "Megafied" by an overdose of "Magna Codex" sat in their arm chairs and slept as unconcerned as Rip Van Winkle, and thrice as long.

With popular sympathy doing so much in their favor for fifty years who would be so foolish as to expect our fat rivals voluntarily to abandon their banner, unless new conditions made it more profitable to do so? Ask a merchant, or a bootblack, or anyone else to desert a lawful occupation that is yielding him a good income, and what will his answer be? "Never, unless something better offers." This one is not paying now, as of yore.

More especially were homeopathy's camp-followers, and the eclectics and the host of irregulars of other creeds who were poorly equipped in the essentials of medicine, satisfied with the partisans and the full purses our "Chinese wall' brought them, and also with the Gibraltar it afforded their defects, against the exposure that consultations and comparison of scientific equipment would bring. This great step on our part will now compel every opponent to stand or fall by what he is, and will force expansion on all among our rivals who can and will expand beyond a limited creed. It will also bring Chinese stagnation to those who cannot or will not, while to all the interests of unfettered medicine it will be like a ladder let down from heaven.

Hahnemann's followers have certainly thrived most where most assailed, as the profession of New York, Philadelphia, Boston, and other parts of New England, Baltimore, Cleveland, and elsewhere can all attest. Now, with this battered target removed, our profession in America will be rid of its greatest hindering weight, and our opponents of today will be gradually transformed into worthy friends, possibly their societies, and colleges, and hospitals, and medical journals into auxiliaries, and we will finally see a union of all true physicians from ocean to ocean. No honest man can compromise on a question of principles, but a consultation, whether with Dr. Newkind, or Dr. Oldkind, is not a question of principle at all, but clearly a matter of humanity and business combined. If we nonsectarians be qualified and honest men, the half we supply in a consultation will be both rational and valuable, and if the half our confreres supply is either erroneous or valueless, it is their fault and not ours.

When we recognise an outsider as a physician it does not require us to endorse either his crotchets or his dogmas, nor has any honest man practising a limited creed anything to fear from meeting one who does not, as truth wherever found has nothing to fear from error. We nonsectarians, with our boundless field of therapeutics, naturally stand on a broader vantage ground and see much to condemn in our opponents, but gentlemen, withal, let us be modest in our claims, for we ourselves are not yet scientifically perfect, our enemies still find much in us for criticism, and there is not a specialist nor a surgeon nor a consultant of any kind anywhere who has not discovered by actual experience that ignorance of this or that well-known medical truth is not confined entirely to irregulars.

As to the moral aspect of mixed consultations: What physician does not come in frequent contact with the vile, the vicious, and the ignoble? Is anyone thereby necessarily contaminated? Not at all! Would it not be equally foolish, or even more so, for anyone to say that if a well-qualified physician gives his advice or his aid to an ignorant one, or even to a medical fool or a knave, and thus aids him in preventing suffering or in saving life, he thereby necessarily descends to that man's level, or degrades our noble calling? To all such assertions I would answer—no. In the name of God No! No!

The occasions will probably always be few in which the patrons of a well-equipped regular physician will ask him to call a sectarian in consultation, but if such a course becomes necessary it should be done without passion or prejudice, and with but one end in view, the good of the patient. After a while, when things change and animosities have died out, we nonsectarians will probably be frequently called to meet every variety of outsiders, and these opportunities for making a more accurate diagnosis, or detecting complications, or pointing out errors, or suggesting better therapeutics, or giving manual aid in difficult cases, or adding good of any kind cannot fail to save thousands of valuable lives, and to show our better equipment and our greater usefulness, and thus nine discerning people will be brought toward us where one will turn away, and the oftener this comparison is made the sooner will all question as to WHICH IS BEST be banished.

How will our change of code affect society membership? According to the estimate of Dr. Simmons, there are today in the United States, 77,000 physicians who for one cause or another, do not belong to medical societies of any kind. Of these each state has its full quota, and one of the benefits that should follow our change of code is an increased membership in this faculty and also in our local societies, because there are numerous persons who are perfectly eligible who doubted their ability to measure up to the code's requirements; others who dissented from this restriction or that feature, and would not affiliate; others knew the code was binding on every member of every society, and would not put themselves under its yoke for fear of unwittingly rendering themselves liable to unfair trial by rivals or enemies; others wished to keep out of code wrangles, and to hold themselves responsible to their own conscience alone, and therefore kept aloof from fellowship; others had no respect for the effete nonintercourse laws of long ago, and would not fraternise under them, as shown twenty years ago by the angry split in the Medical Society of the State of New York. Now, after this great and mollifying change of code we ought to have improved unity everywhere, and see increased membership. Where is the Welch, or the Osler, or the Ashby, who will essay this task among those who are delinquent in Maryland?

There will be another important result: the code has for years kept many who hold but loosely to similia from making its therapeutic bugaboo a secondary matter, and entering our broader nonsectarian field. We all know that the majority of the prominent men who are now known as homeopaths are much more lukewarm toward its restricting tenets than their predessors were, because their education, talents and dispositions are too liberal to remain confined to the limits of any one creed, and many of these will now gradually throw off their sectarian shackles and finally be found enjoying the wider field with us, because they are no longer ostracised, the finger is no longer pointed, and the cry of "outlaw" is no longer heard. Indeed, we and their more scientific representatives are even now not nearly so far apart as many suppose. At the annual meeting of the American Institute of Homeopathy, held at Cleveland, Ohio, June, 1902, in a learned annual address on "The Present Status of Homeopathy," the eminent president, Professor James C. Wood, after enumerating seven different classes of cases, each presenting indications for therapeutics, which he admits Homeopathy does not reach, concludes by saying: "The legitimate sphere of action of homeopathy is—the curing of diseases which are curable by the principle of substitution."

To put it all in a paragraph: the president of the chief homeopathic medical body of America, divides the structure of therapeutics into numerous stones and merely claims that similia is one of these, not even that it is the foundation stone, not even that it is the keystone, but only that it is one of the stones. I ask in the name of Hippocrates, what regular physician on earth would object to his next door neighbor, or even to his own brother, for holding these tenets if he wishes, or for following this, that or the other therapeutic line he chooses; but were he our brother we would probably lay our hands gently on his shoulder, and beg him for humanity's sake not to chain himself to any one segment of the therapeutic circle.

The educated homeopaths of today differ radically from their predecessors in numerous ways and are now shoulder to shoulder with us everywhere, in obtaining and maintaining salutary medical laws to shut out ignorance and quackery, and are fast approaching us in many other respects. The truth is, the chief difference between the majority of their 9,369 and us today, is not the question of therapeutics, but merely in name and affiliation, and I firmly believe an honest show of liberality on our part will do much to make this disappear, and to cause many to drop their dogmatic titles and throw off their sectarian chains and enter the broader and nobler sphere expressed all over God's world by the single word, *physician.*

Every thoughtful son of Esculapius knows that true medicine is an unfettered profession, therefore while we earnestly desire unity with all worthy men, our mountain cannot go to anybody's Mohammed. The reason why is well expressed in Section I, Article 1, of Chapter II of our "Principles of Medical Ethics," which says: "It is inconsistent with the principles of medical science, and it is incompatible with honorable standing in the medical profession for physicians to designate their practice as based on an exclusive dogma or a sectarian system of medicine." Let us be liberal in all else and this broader and better course in therapeutics will finally be adopted by all.

I am neither an enthusiast nor a rainbow chaser, nor do I believe abolishing the code will at once bring prodigious results, but as sure as day follows night, results great and good will come, and those who are working in the Esculapian field 15 or 20 years hence will see the great benefit that will result to us and to them from the passing of the conditions created by our old code. The American Medical Association has left to the state organisations discretionary powers in deciding who are, and who are not, eligible to membership in them, and in their subordinate societies. It also leaves them the right to adjudicate for themselves all other ethical questions, and among the first duties now before us is promptly to put the Medical and Chirurgical Faculty of Maryland in line with our great national body.

Finally, the sooner you and I, and every other physician in America do our part to let the whole world know that our profession has changed the tomahawk for the olive branch, the sooner will we create a vastly improved public opinion, and the sooner will we enter on a long era of peace, good will and prosperity.

But medicine is a diversified profession, and in its rank are naturally found, many men of many minds, and no reasoning person can expect all to agree fully that it was judicious to change our venerated grandfather's code, and some may even think it was a huge mistake, but, after studying the whole subject, I believe that it was an exceedingly wise act, and, I humbly predict that it will have at least 5 excellent results: it will free us from the old charge of bigotry; it will kill the false cry of persecution; it will have a great and far-reaching effect on all our material interests; it will everywhere promote and foster professional unity; and, far above all else, by putting an end to partisan agitations it will increase the good repute of every worthy medical man in America.

